# Evaluation of Ecological Suitability and Quality Suitability of *Panax notoginseng* Under Multi-Regionalization Modeling Theory

**DOI:** 10.3389/fpls.2022.818376

**Published:** 2022-04-28

**Authors:** JiaQi Yue, ZhiMin Li, ZhiTian Zuo, YuanZhong Wang

**Affiliations:** Medicinal Plants Research Institute, Yunnan Academy of Agricultural Sciences, Kunming, China

**Keywords:** *Panax notoginseng*, maximum entropy model, ecological suitability, quality suitability, multi-regionalization modeling

## Abstract

*Panax notoginseng* is an important medicinal plant in China, but there are some limitations in the ecological suitability study, such as incomplete investigation of species distribution, single regionalization modeling, and lack of collaborative evaluation of ecological suitability, and quality suitability. In this study, the maximum entropy model was used to analyze the ecological suitability of *P. notoginseng* under current and future climates. The multi-source chemical information of samples was collected to evaluate the uniformity between quality and ecology. The results showed that the current suitable habitat was mainly in southwest China. In the future climate scenarios, the high suitable habitat will be severely degraded. Modeling based on different regionalization could predict larger suitable habitat areas. The samples in the high suitable habitat had both quality suitability and ecological suitability, and the accumulation of chemical components had different responses to different environmental factors. Two-dimensional correlation spectroscopy combined with deep learning could achieve rapid identification of samples from different suitable habitats. In conclusion, global warming is not conducive to the distribution and spread of *P. notoginseng*. The high suitable habitat was conducive to the cultivation of high-quality medicinal materials. Actual regionalization modeling had more guiding significance for the selection of suitable habitats in a small area. The multi-regionalization modeling theory proposed in this study could provide a new perspective for the ecological suitability study of similar medicinal plants. The results provided a reference for the introduction and cultivation, and lay the foundation for the scientific and standardized production of high-quality *P. notoginseng*.

## Introduction

*Panax notoginseng* (Burk.) F. H. Chen is a perennial herb of *Panax* genus, whose dry root and rhizome are valuable medicinal herbs, peculiar to China. Modern studies have proved that *P. notoginseng* mainly contains saponins, flavonoids, volatile oils, sterols, organic acids, polysaccharides, amino acids, and other chemical components (Wang et al., 2006, 2016, 2020; Li et al., 2013; Liu et al., 2020), and has anti-inflammatory, anti-tumor, protecting the cardiovascular system, improving the immunity and blood circulation and inhibiting the metastasis of liver tumor cells and other pharmacological effects (He et al., 2012; Zhou et al., 2014; Xie et al., 2018; My et al., 2021). Due to the uniqueness of the growth environment, *P. notoginseng* has become the dominant medicinal material in China. As the main raw material of traditional Chinese medicine preparation, decoction pieces, and Chinese patent medicine, it is also an important pillar for the development of the regional economy, which is of vital importance to the development of the traditional Chinese medicine industry and even the whole health industry (Cui et al., 2014). However, *P. notoginseng* is a typical ecologically fragile shade plant with a narrow distribution area. Coupled with problems, such as continuous cropping obstacles, diseases, insect pests, and other problems, the origin of *P. notoginseng* in Wenshan in Yunnan faces a planting bottleneck (Ou et al., 2021). In addition, there are reports of deforestation to plant *P. notoginseng* in 14 prefectures and cities in Yunnan, which poses varying degrees of harm to forest resources and the ecological environment (YUNNAN.CN, 2019). The contradiction between the protection of forest resources and the standardized development of *P. notoginseng* planting industry is becoming increasingly prominent. Accordingly, there is an urgent need for the introduction and expansion of new planting areas.

*Panax notoginseng* is an economic medicinal plant that has been cultivated and domesticated earlier. It has been artificially cultivated for more than 400 years, and its demand has been increasing sharply year after year (Wang et al., 2013). Traditionally, *P. notoginseng* in Wenshan has the best quality and effect. In order to meet the market demand, new planting areas have been developed in Kunming, Qujing, Honghe, and other regions. The quality differences in different regions reflect that the growing development of *P. notoginseng* is affected by natural ecological factors (Tao and Wu, 2003). Therefore, blind introduction and expansion will seriously affect the rational distribution of *P. notoginseng* production, greatly weakening the authenticity of medicinal materials, resulting in a serious decline in the quality of medicinal materials. When planting, it is extremely important to determine the suitable area for medicinal materials. For thousands of years, the evaluation of high-quality medicinal materials has been limited to the relationship between the efficacy and place of production. The understanding of the relationship between medicinal materials and the environment is still vague and superficial, failing to reveal its intrinsic relevance. The research on the ecology of medicinal plants and the ecology of traditional Chinese medicine resources should be actively strengthened to realize the synergy between the ecological suitability and quality suitability of medicinal plants.

Existing studies have analyzed the ecological suitability and quality ecology of *P. notoginseng* to different degrees. The possible distribution of the ecological suitable area and the main ecological factors affecting the geographical distribution were discussed (Zhang H.Z., et al., 2016). The study indicated that the possible ecological suitable areas in China were distributed in Yunnan, Guangxi, Guangdong, Guizhou, Hainan, Sichuan, Fujian, and Chongqing, accounting for more than 70% of the world's optimum cultivation area. Yunnan and Guangxi were the most suitable cultivation areas. The United States, Brazil, Portugal, and other countries also have a small number of optimal cultivation areas (Meng et al., 2016). The main ecological factors affecting the geographical distribution of *P. notoginseng* are the precipitation of warmest quarter, temperature seasonality, altitude, isothermality, coefficient of variation of precipitation seasonality, the mean of monthly, and the precipitation of the driest month (Zhang Q. et al., 2016). Although current studies can provide a theoretical basis for the suitable regionalization of *P. notoginseng*, there are some limitations, such as incomplete investigation of species distribution, single regionalization modeling method, and lack of ecological and quality coordination research, which seriously restrict the reliability of research results. In addition, most regionalization studies aimed at suitability accounted for the majority, while few regionalization studies focused on the accumulation of secondary metabolites. The regionalization studies did not discuss whether the growth suitability and quality suitability of the suitable area was consistent, resulting in that the suitable area obtained had no practical value for *P. notoginseng* cultivation, land use, and social economy.

The present study conducted a more comprehensive investigation and collection of the actual distribution of *P. notoginseng*. At the theoretical level, the interaction between the environment and planting of *P. notoginseng* was analyzed to find the key ecological factors, and the maximum entropy (MaxEnt) model was used to explore the potentially suitable habitat and simulate the impact of climate change on the distribution of *P. notoginseng* in the future. At the practical level, modern analytical methods were used to integrate multi-source chemical information of *P. notoginseng* samples, analyze the ecological suitability regionalization and quality suitability regionalization collaboratively, and establish a rapid identification method of *P. notoginseng* from high-quality suitable habitat. It is proposed to combine theory and practice on the basis of current species distribution, reveal the geographical distribution pattern of *P. notoginseng* under current and future climate scenarios, and evaluate the consistency of ecological suitability regionalization and quality suitability regionalization. In this way, the quality of the source is guaranteed, and a scientific basis is provided for the selection of suitable planting regionalization for *P. notoginseng* under future climate change.

## Materials and Methods

### Species Distribution Data

The distribution data of *P. notoginseng* were obtained by three ways: (1) From 2011 to 2018, the Institute of Medicinal Plants, Yunnan Academy of Agricultural Sciences conducted field surveys on the major distribution areas in Yunnan, Guangxi, and surrounding areas, recorded the longitude and latitude information of planting sites in detail, and compiled data sets of species distribution. (2) Species distribution information was obtained through literature review and online sharing platform of specimen digital information. Platforms include the Chinese Virtual Herbarium databases of National Plant Specimen Resource Center (CVH, https://www.cvh.ac.cn), the National Specimen Information Infrastructure (NSII, www.nsii.org.cn), and the Global Biodiversity Information Facility (GBIF, https://www.gbif.org). (3) Government news reports combined with Google satellite images conducted investigation and image analysis of greenhouses for planting *P. notoginseng* in Yunnan, Guangxi, Guizhou, Sichuan, and in Chongqing from 2020 to 2021, so as to supplement and improve the species distribution data set. Through the integration of the above data sets, 3,475 species distribution data were obtained.

**Figure 1:**
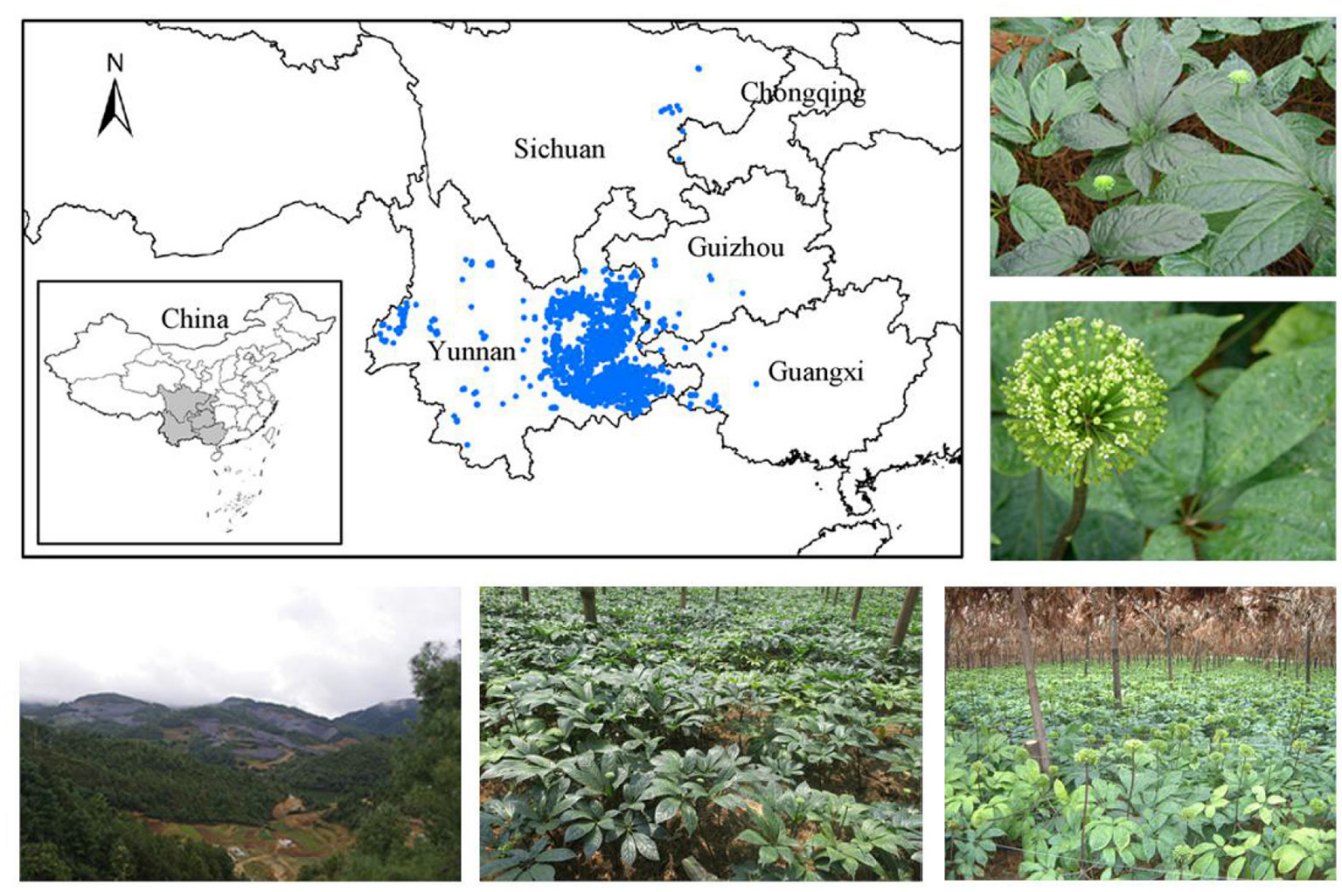
The picture information on 2,122 distribution records, habitats, and samples of *Panax notoginseng*.

The species distribution data were further filtered and screened. First, the items with duplicate origin information and incomplete latitude and longitude information were removed. Then, based on Google Earth and the latitude and longitude information of specimens, the entries with incorrect origin information were revised or deleted. Finally, in order to avoid the redundancy of distribution data during modeling, ArcGIS was used to buffer and analyze species distribution data, and repeated data within 1 km was deleted to ensure that there was only one species distribution point in each 1.0 × 1.0 km^2^ grid. Through the above screening, 2,122 species distribution data (Figure 1) were finally reserved for MaxEnt modeling analysis. Details of the distribution data are shown in Supplementary Table S1.

### Environmental Variables

The environment variables for modeling mainly include elevation data and bioclimatic variables (Table 1). All these variables (spatial resolution of 30 s and raster data of about 1.0 × 1.0 km^2^) were downloaded from the WorldClim (https://www.worldclim.org/). The climate variables required to study current species distributions were obtained from the grid data generated by interpolating observations from global climate stations from 1970 to 2000. Future climate data were predicted by the medium-resolution Climate System Model (BBC-CSM2-MR) developed by the National Climate Center (Beijing). Two greenhouse gas concentration pathways (medium-low concentration scenario: SSP245 and high concentration scenario: SSP585) under two future periods (2050s: 2041–2060 and 2090s: 2081–2100) were selected to study. The precision of future climate data provided by WorldClim is 2.5 min. In order to facilitate modeling, the data of spatial resolution was unified at 30 s by using ArcGIS raster resampling.

**Table 1:** The environment variables information.

**Type**	**Abbreviation**	**Environment variables**	**Unit**
Climate factor	Bio01	Annual mean temperature	°C
	Bio02^*^	Mean diurnal range	°C
	Bio03^*^	Isothermality	/
	Bio04^*^	Temperature seasonality	°C
	Bio05	Max temperature of warmest month	°C
	Bio06	Min temperature of coldest month	°C
	Bio07^*^	Temperature annual range	°C
	Bio08	Mean temperature of wettest quarter	°C
	Bio09	Mean temperature of driest quarter	°C
	Bio10	Mean temperature of warmest quarter	°C
	Bio11	Mean temperature of coldest quarter	°C
	Bio12	Annual precipitation	mm
	Bio13^*^	Precipitation of wettest month	mm
	Bio14^*^	Precipitation of driest month	mm
	Bio15^*^	Precipitation seasonality	/
	Bio16	Precipitation of wettest quarter	mm
	Bio17^*^	Precipitation of driest quarter	mm
	Bio18^*^	Precipitation of warmest quarter	mm
	Bio19^*^	Precipitation of coldest quarter	mm
Topographic factor	Ele^*^	Elevation	m

*Variables with an asterisk*

(*) *were used for the final modeling*.

In order to avoid over-fitting of modeling results due to multicollinearity among environmental variables, elevation and bioclimatic variables corresponding to species distribution data were extracted for Spearman's correlation analysis. Combined with the modeling results, when the variables are significantly correlated and the correlation coefficient is >0.8, the variables with small modeling contributions are eliminated. All environment variables were converted to ASCII format before modeling. The final variables used for modeling are shown in Table 1.

### Model Establishment and Evaluation

#### Regionalization of Modeling

Regionalization, which means the division of regions, is a way for people to extract spatial information according to a specific purpose, and to realize the understanding of the natural or the social environment through the region-based classification and merger (Margules and Pressey, 2000; Margules et al., 2002). The regionalization of traditional Chinese medicine resources is to study the spatial differentiation law of traditional Chinese medicine resources and their regional systems, and to divide them into regions according to this spatial consistency and difference law (Chinese Medical Company, 1995; Zhu et al., 2017). The traditional method of modeling based on all distribution points (i.e., overall regionalization) may have poor representativeness and ignore the internal relationship between the quality of medicinal materials and the regional ecological environment. As a result, the model results are only of theoretical significance and lack of practical guiding significance combined with actual regionalization. Through the comprehensive utilization of more reliable and reasonable species regionalization results, the collaborative modeling of ecological suitability regionalization and production suitability regionalization can greatly improve the practicability of predicted suitable habitat for *P. notoginseng*. Therefore, in addition to modeling for overall regionalization, this study also divided all distribution sites into the following regionalization according to the topographic characteristics of the distribution region, the actual planting situation, the authenticity of planting region, the traditional production area, the new production area, and other restrictions: (1) Hengduan Mountains, (2) Yunnan-Guizhou Plateau, (3) Yunnan + Guangxi, (4) Sichuan Basin, (5) Sichuan + Chongqing + Guizhou, and (6) Sichuan + Chongqing + Guizhou + Guangxi + Wenshan. It is planned to compare and predict the suitable habitat areas of *P. notoginseng* through different regionalization modeling.

#### Parameter Setting and Establishment of Model

MaxEnt modeling is one of the most widely used algorithms in the recent years (Phillips et al., 2006). This method has been widely used in the prediction of geographic distribution of medicinal plant species with high simulation accuracy (Guisan and Zimmermann, 2000; Elith et al., 2011). In this study, species distribution data in CSV format and environment variable data in ASCII format were imported into MaxEnt software (Version 3.4.4). About 75% of distribution data were set as training set and the remaining 25% as test set. Maximum iterations were set to 5,000, and the remaining parameters were default values. Model prediction results were saved as ASCII files.

The area under the curve (AUC) value of the area under the receiver operating characteristics (ROC) curve was used to evaluate the accuracy of the model results. The AUC value ranges from 0 to 1, and the closer the value is to 1, the more reliable the prediction results are (Hanley and McNeil, 1982; Zweig and Campbell, 1993). The index of important variables affecting the species distribution was determined by Jackknife test combined with the cumulative contribution rate of modeling environmental variables (Phillips, 2005).

The ASCII files obtained by MaxEnt operation were imported into ArcGIS and converted into raster data. Raster data were reclassified according to habitat suitability index (HSI). The suitability was divided into four categories according to the difference of suitability index: high suitability (HIS ≥ 0.6), moderate suitability (0.4 ≤ HIS < 0.6), low suitability (0.2 ≤ HIS < 0.4), and no suitability (HIS < 0.2) (Shen et al., 2021). The areas of various suitable habitat were calculated by Zonal Statistics of ArcGIS.

### Post-processing of Model Results

The spatial extent of suitable habitat of *P. notoginseng* was modeled based on the current climate data. Since the overall regionalization is more representative of the actual distribution of *P. notoginseng*, the modeling projections of two future periods (2050s and 2090s) and two emission scenarios (SSP245 and SSP585) were performed based on the overall regionalization. Then, the HIS for current and future suitable habitats were calculated based on the results of MaxEnt software (probability of species presence). According to the suitability index, the habitat suitability of *P. notoginseng* was divided into high suitability, moderate suitability, low suitability and no suitability.

The changes of current and future suitable habitats were evaluated by calculating the suitable habitat change rate (SHCR) of high suitability, moderate suitability, and low suitability.


$$\begin{array}{l} SHCR=\;\left(AF-AC\right)/AC. \end{array}$$

### Ecological Quality Suitability Analysis of *P. notoginseng*

#### Sample Collection

At present, the cultivation area of *P. notoginseng* is mainly concentrated in Yunnan, China. In addition, according to the survey results of actual distribution sites, there are more distribution sites in the five regions of Wenshan, Honghe, Yuxi, Kunming, and Qujing in Yunnan Province. Therefore, considering the actual planting situation, the economic value of production and the prediction results of suitable habitat for the Maxent model, these five regions were selected for sampling, and a total of 455 samples of *P. notoginseng* were collected. The picture information of some habitats, collection sites, and samples are shown in Figure 1. All samples were identified as *P. notoginseng* (Burk.) F. H. Chen by Professor JinYu Zhang, Institute of Medicinal Plants, Yunnan Academy of Agricultural Sciences. After sampling, the roots of fresh samples were taken, cleaned, and dried in a constant temperature oven at 50°C. Then all samples were crushed with a grinder, screened through 80 mesh, and stored in ziplock bags to avoid light for subsequent analysis. The number of samples used for content analysis and spectral analysis is shown in Supplementary Table S1.

#### Content Determination

In this study, the contents of notoginsenoside R_1_, ginsenoside Rg_1_, ginsenoside Re, and ginsenoside Rb_1_ were determined by high-performance liquid chromatography (HPLC) analysis. The contents of total flavonoids and total polysaccharide were determined by UV-visible spectrophotometry. Chromatographic analyses were performed with LC-10 ATvp liquid chromatograph system with a diode array detector. binary pump, manual sampler, and CLASS-VP workstation. The chromatographic separation was achieved using an Inertsil ODS-3 column (4.6 × 150 mm, 5 μm). Standards of notoginsenoside R_1_, ginsenoside Rg_1_, ginsenoside Re, ginsenoside Rb_1_, and rutin were purchased from the Chinese National Institute for Food and Drug Control (Beijing, China). Anhydrous glucose was purchased from Sigma-Aldrich. HPLC grade acetonitrile and methanol were purchased from Thermo Fisher Scientific (Fair Lawn, NJ, USA). Other chemicals and reagents were of analytical grade.

Determination of four kinds of saponins, such as Notoginsenoside R_1_, ginsenoside Rg_1_, ginsenoside Re, and ginsenoside Rb_1_ were precisely measured at 2.00, 3.00, 2.20, and 2.40 mg, respectively, and dissolved in 1 mL of methanol solution. The solution was diluted 7 times according to the gradient of 60% to obtain a total of 8 mass concentration gradients of the reference solution. Sample powder was weighed accurately to 100 ± 0.30 mg and extracted with 2.0 mL of methanol by an ultrasound-assisted method for 60 min at ambient temperature. The methanol loss was supplemented after ultrasound, and the extract solution was filtered using a 0.22 μm membrane filter, which was the solution to be tested. The mobile phase and gradient elution procedures refer to HPLC detection of *P. notoginsen*g in the Chinese Pharmacopeia (I division, 2015 edition) (National Pharmacopoeia Committee, 2015). The mobile phase consisted of ultrapure water (A) and acetonitrile (B). The gradient elution sequence was conducted as follows: 0–12 min (19% B), 12–60 min (19–36% B). The column temperature was 30°C. The flow rate was kept at 1 mL/min and the injection volume was 10 μL. Detective wavelengths were set at 203 nm (Ma, 2014).

Determination of total flavonoid: Rutin was weighed accurately to 1.20 mg, dissolved in 8 mL of 70% ethanol solution, and then added 0.1 mol·L^−1^ of AlCl_3_ ethanol solution (chromogenic agent) to 10 mL. The mother liquor of standard substance was diluted 6 times according to the equal gradient of 60% to obtain the standard substance solution with seven concentration gradients. Sample powder of 50 ± 0.2 mg were accurately weighed, and 8 mL of 70% ethanol solution was added for ultrasonic extraction for 1 h. Then, 6 mL of extracts were absorbed, and color reagent was added to 8 mL. Color developing time was 20 min, and scanning range was 190–600 nm. The background was scanned using a blank solution with chromogenic agent before collecting the sample spectra (Sun et al., 2018).

Determination of total polysaccharides: Anhydrous glucose was accurately weighed to be 30.50 mg, and the volume was adjusted to 100 mL with ultrapure water. The sample powder of 500.0 ± 1.0 mg was accurately weighed, and the volume was fixed to 50 mL with ultrapure water. Ultrasonic extraction was performed at 60°C water bath for 2 h, and the lost solution was supplemented after completion. The extract was quickly filtered by a quantitative filter paper. After the filtrate was cooled to room temperature, 20 mL of filtrate was absorbed into a 50 mL centrifuge tube. After heating, the filtrate was volatilized to 10 mL, and then 100% ethanol was added to the filtrate at a constant volume of 50 mL. After blending, the filtrate was left to settle for 12 h. After precipitation, the solution was centrifuged at a speed of 9,000 r/min for 10 min, and the supernatant was extracted after centrifugation. The precipitation was washed with 30 mL of 80% ethanol solution and centrifuged at a speed of 9,000 r/min for 5 min. The supernatant was extracted and washed twice. The precipitation was added with ultra-pure water at a constant volume of 40 mL, and then heated in boiling water bath for 30 min after uniform oscillation, which was the solution to be tested. The glucose standard solution of 0.05, 0.1, 0.2, 0.4, 0.6, 0.8, and 1.0 mL was accurately pipetted into test tubes, and water is added to 2 mL, respectively. About 1.0 mL of the sample solution was precisely pipetted into a test tube, water was added to 2 mL, and finally, 1 mL of 5% phenol solution was added. After shaking, 4 mL of concentrated sulfuric acid was slowly added along the wall of the test tube, shaken quickly, and heated in a boiling water bath for 30 min. The solution was cooled to room temperature, and the UV scan range was set to 350–600 nm. The background was scanned using a blank solution with chromogenic agent before collecting the sample spectra (Li et al., 2017).

#### Fourier Transform Mid-infrared and Near Infrared Spectra Acquisition

The Fourier transform mid-infrared spectra (FT-MIR) of each samples were performed by the Fourier transform infrared spectrometer (Perkin Elmer, Norwalk, CT, USA) equipped with a deuterated triglycine sulfate (DTGS) detector. The sample powder (1.2 ± 0.2 mg) and KBr powder (15.0 ± 1.0 mg) were accurately weighed and mixed in an agate mortar. After grinding, the mixture was poured into a mold and pressed to make uniform flakes. Each sample tablet was tested to obtain FT-MIR spectra that was recorded in the region of 4,000–650 cm^−1^ with a resolution of 4 cm^−1^, and a total of 16 scans were performed. Two scans were repeated for all samples to obtain an average spectrum. Prior to sample scanning, the background was scanned using blank KBr tablet to remove carbon dioxide and water interference.

The near infrared spectra (NIR) of sample powder were collected with an Antaris II spectrometer (Thermo Fisher Scientific, USA) equipped with an integrating sphere diffused reflection mode and Result 2.1 software. The wavenumber region was set to 10,000–4,000 cm^−1^, and the resolution was set to 8 cm^−1^. About 64 scans were accumulated for a single sample, and each sample was repeated for three times to obtain the average spectrum. The spectral record after acquisition is the logarithm of reciprocal reflectance.

#### Analysis of Content Data

To investigate whether *P. notoginseng* samples with ecological suitability (these samples are usually from highly suitable habitat areas) has quality suitability, the contents of notoginsenoside R_1_, ginsenoside Rg_1_, ginsenoside Re, ginsenoside Rb_1_, total flavonoids, and total polysaccharides in these samples were compared and analyzed. In addition, we drew the boxplot for intuitively displaying the content data distribution characteristics of *P. notoginseng* samples from Wenshan, Honghe, Yuxi, Kunming, and Qujing.

Principal component analysis (PCA) is an unsupervised pattern recognition method, which transforms multiple related variables into a group of unrelated new variables by dimensionality reduction, while preserving the original sample information as much as possible (Abdi and Williams, 2010). In this study, simca software was used to conduct PCA on the content data and 20 environmental variables of five regions, in order to explore the similarity and differentiation of environmental variables, and the correlation between environmental variables and content.

#### Discriminant Evaluation Based on Deep Learning

Deep learning is the main research method adopted in the development of artificial intelligence at the present stage, which has unique advantages in image classification and object recognition (LeCun et al., 2015). The spectroscopy technology has the advantages of fast, lossless, and convenient. In particular, a two-dimensional correlation spectroscopy (2DCOS), which has the advantages of multi-discipline, can greatly improve the resolution of spectra through the increase of dimension, and enrich the information carried by the spectra (Noda, 1989). Combining it with deep learning for the identification of medicinal plants can give full play to the advantages of the two technologies and greatly improve the efficiency of identification analysis. In this study, a deep learning method combined with 2DCOS was developed to rapidly identify samples from different suitable habitat areas.

Generalized 2DCOS is an effective method to improve the spectral resolution and solve spectral overlap problem by designing interference variables, which is obtained by discrete generalized two-dimensional correlation spectral algorithm. Its dynamic spectra are expressed as *S*, and the expression is provided in Equation (1), where *v* is the variable and *t* is the external disturbance (Noda, 1993).


(1) $$\begin{array}{l} S\left(v\right)\;=\;\left[\begin{array}{c} y\left(v,t_{1}\right)\\ y\left(v,t_{2}\right)\\ y\left(v,t_{3}\right)\\ ▪\\ ▪\\ ▪\\ y\left(v,t_{m}\right) \end{array}\right] \end{array}$$

Studies have shown that synchronous 2DCOS is more suitable for the identification of medicinal plants (Yue et al., 2021). Therefore, we combined it with deep learning to identify samples from different suitable areas. The synchronous spectral intensity is, and its expression is provided in Equation (2).


(2) $$\begin{array}{l} \Phi \;\left(v_{1},v_{2}\right)\;=\;\frac{1}{m-1}S\;{\left(v_{1}\right)}^{T}\cdot S\left(v_{2}\right) \end{array}$$

Matlab2017b software was used to automatically generate synchronous 2DCOS images. All images were stored in the corresponding folder in the JPEG image format of 64 × 64-pixel size, which is used to build the ResNet model. All data sets were divided into training set (60%), test set (30%), and external validation set (10%) using the Kennard–Stone algorithm.

In this study, a 12-layer ResNet was established with a weight attenuation coefficient λ of 0.0001 and a learning rate of 0.01. Supplementary Table S2 showed the ResNet network parameter configuration. The model structure is shown in Supplementary Figure S1, where the input data is synchronous 2DCOS images. The identification flow chart of ResNet is shown in Supplementary Figure S2. The training set was used to train the model. The Stochastic Gradient Descent (SGD) method was adopted to search for optimal parameters and minimize the loss function value, so as to obtain the optimal model. The test set was used to verify whether the performance of the final model was optimal. The external validation set was used to verify the generalization ability of the model.

## Results and Discussion

### Model Evaluation and Important Environmental Variables

The AUC value can be used to evaluate the credibility and accuracy of the model results, and its value is between −1 and 1. For the AUC value, 0.60 < AUC ≤ 0.70 indicates that the model effect is fair, the value 0.70 < AUC ≤ 0.80 indicates that the model effect is good, the value 0.80 < AUC ≤ 0.90 indicates that the model effect is very good, and the value 0.90 < AUC ≤ 1.00 indicates that the model effect is excellent (Swets, 1988). The AUC values of the prediction results of the seven models were all higher than 0.92, indicating that all models had excellent fitting effects and high prediction accuracy.

Eleven climate factors were selected as indicators to simulate the suitable habitat of *P. notoginseng*, and the main climate factors affecting the distribution of *P. notoginseng* were obtained. Supplementary Table S3 shows AUC values and important environmental variables of the seven regionalization models under current climate conditions. The results of important environmental variables were obtained from the comprehensive analysis of contribution analysis of variables and the jackknife test of variable importance, which were listed in the descending order of importance as follows. The variables that had a higher contribution to the overall regionalization model were Bio04, Bio07, Ele, and Bio17, and their cumulative contribution rate was 95.32%. For the Hengduan Mountain regionalization model, Bio04, Bio03, Bio14, Bio17, and Ele had a high contribution rate to the potential distribution of *P. notoginseng* with a cumulative contribution rate of 94.08%. The variables of Bio04, Ele, Bio02, Bio17, and Bio14 contributed significantly to the potential distribution of *P. notoginseng* in Yunnan-Guizhou Plateau regionalization model, with a cumulative contribution rate of 93.40%. For Yunnan + Guangxi regionalization model, Bio04, Bio07, and Bio17 contributed significantly to the potential distribution, with a cumulative contribution rate of 96.48%. The variables of Bio04, Ele, Bio03, and Bio19 had a high contribution rate to the potential distribution in the Sichuan Basin regionalization model, with a cumulative contribution rate of 100.00%. Bio18, Bio14, Bio15, Bio02, Ele, and Bio19 had a high contribution rate to the potential distribution of *P. notoginseng* in the Sichuan + Guizhou regionalization model, with a cumulative contribution rate of 93.84%. For Sichuan + Guizhou + Guangxi + Wenshan regionalization model, Bio17, Bio04, Ele, Bio13, Bio15, and Bio07 contributed significantly to the potential distribution of *P. notoginseng* with a cumulative contribution rate of 99.53%.

In summary, there are certain differences in the important environmental variables affecting the distribution of suitable habitats in different regions, which has reference significance for the introduction and cultivation of *P. notoginseng* in different regions. On the whole, the main ecological factors affecting the spatial distribution of *P. notoginseng* are Bio04, Ele, and Bio17. According to relevant literature reports, the elevation of the most suitable planting area of *P. notoginseng* is about 1,400–1,800 m, and the incidence of black spot will increase with the higher elevation beyond the suitable range (Dong et al., 2016). In addition, precipitation and temperature have a vital influence on the growth and distribution. *P. notoginseng* prefers humidity and warmth, but excessive high temperatures and excessive precipitation during the growth period will cause diseases to occur, and too little will not meet the growth needs (Zhang Q. et al., 2016). Therefore, special attention should be paid to the reasonable control of moisture and temperature during the cultivation and introduction process in different regions. From the similarity of important environmental variables, Models 2, 3, 6, and 7 are relatively similar, while Models 4 and 5 are significantly different. The results showed that there was a certain correlation between the existing ecological environment of different regionalization, which could provide a certain reference for the division of real regions, so that the regionalization results could be truly used in the production of *P. notoginseng*. In addition, similar climate is a prerequisite for introduction and cultivation, and it is more likely to expand *P. notoginseng* successfully in regionalization with high similarity to traditional producing areas.

### Current Distribution of *P. notoginseng* Under Overall Regionalization Model

**Figure 2:**
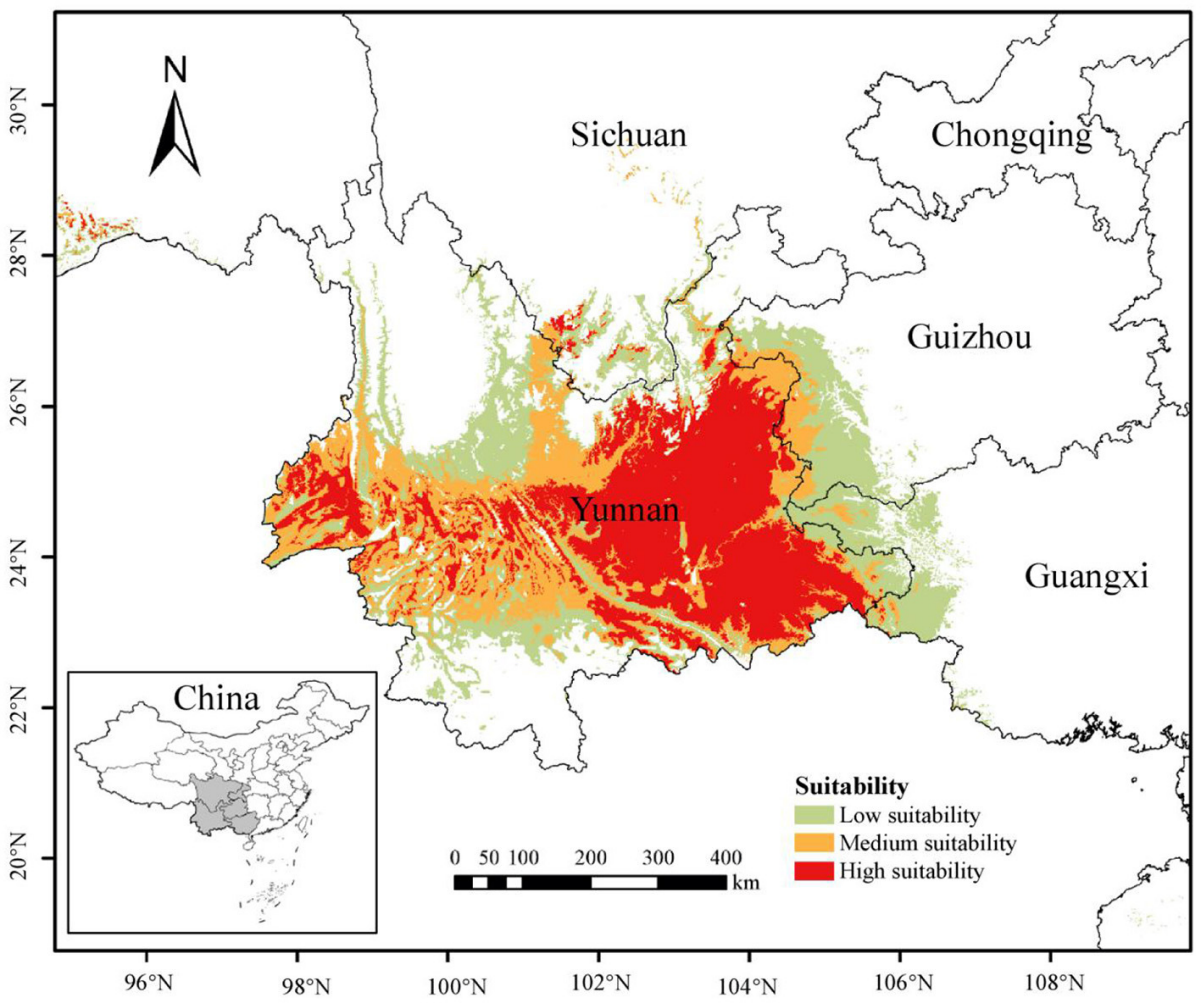
The potential distribution of *Panax notoginseng* under current climate based on the overall regionalization model.

The AUC values of training set and test set data of MaxEnt model under the overall regionalization were 0.927 (Supplementary Table S3), indicating that the model has excellent fitting effect and high prediction accuracy. Based on MaxEnt prediction results, the potential distribution of *P. notoginseng* under the overall regionalization model was divided and visualized, as shown in Figure 2. The suitable habitat distribution of *P. notoginseng* under current climatic conditions was mainly in the southwest of China. Highly suitable habitat areas were concentrated in Yunnan Province, presenting a horizontal distribution with broad areas in the east and sparse areas in the west. In the western margin of Guangxi, the southwest margin of Guizhou, and the southern margin of Sichuan, there are highly suitable habitat areas with small area. The high suitable habitat in Yunnan were mainly distributed in the central and southeast Yunnan, including Wenshan, Honghe, Qujing, Kunming, Yuxi, and Chuxiong. It is also distributed in some prefectures and cities of west and south Yunnan, such as Baoshan, Lincang, Dehong, and Pu'er. The moderate and low suitable habitat mainly include the central part of Hengduan Mountain region, the northwest part of Yunnan-Guizhou Plateau, and the borderlands of Yunnan, Guizhou, Guangxi, and Sichuan Province. Currently, the total area of suitable habitat in China is 27.85 × 10^4^ km^2^, of which the area of high suitable habitat is 10.01 × 10^4^ km^2^, the area of moderate suitable habitat is 8.44 × 10^4^ km^2^, and the area of low suitable habitat is 9.40 × 10^4^ km^2^ (Supplementary Table S4). The area of high suitable habitat in Yunnan is 9.74 × 10^4^ km^2^, accounting for 97.30% of that in China.

The model results show that the potential distribution area of *P. notoginseng* is relatively limited, which is consistent with the actual distribution of *P. notoginseng*. The main reason for this result is that *P. notoginseng* has special requirements on climate, soil, vegetation, and other growth environment (Zhang H.Z., et al., 2016). However, due to the rapid increase in the demand for *P. notoginseng* and the shortage of suitable land resources, the planting range has been expanded to many Prefectures and cities of Yunnan, Guangxi, Guizhou, Sichuan, and other places (Li et al., 2018). From the actual distribution and model prediction results, we can find that although there are suitable habitats in the northwestern Yunnan, *P. notoginseng* has not been planted on a large scale as in the southeastern Yunnan. We speculate that the accumulation of temperature and humidity in northwestern Yunnan is lower than that in the southeastern Yunnan since the barrier of Ailao Mountain is not conducive for the growth of *P. notoginseng*. In addition, the terrain in the northwestern Yunnan is steep. In terms of regional topography and geomorphology, this may also be one of the reasons why *P. notoginseng* has not been planted in large areas in the northwestern Yunnan. On the contrary, why Sichuan, as a new production area, can successfully plant *P. notoginseng* is also a question worthy of discussion and of great practical significance for the expansion of *P. notoginseng*. Judging from the distribution sites in Sichuan, almost all the planting sites are close to the river, which provides a guarantee for the important humidity conditions required for the growth of *P. notoginseng*. We speculate that this is one of the factors influencing its successful planting. Second, the accumulated temperature of Sichuan is similar to that of Yunnan, which may also be an important influencing factor. In future, the production of *P. notoginseng* will face the problem of further expansion of the production area and re-selection of the location. The current overall regionalization modeling is a general prediction of the suitable habitat, which has certain significance for actual planting. However, Chinese medicinal materials have spatial consistency and differences in climate, topography, landform, vegetation, soil, and other aspects according to natural conditions. Discussions on the suitability of Chinese medicinal materials based on regionalization may have greater reference value for actual production, planting, and quality. Therefore, it is necessary to explore suitable habitats for *P. notoginseng* under different regionalization according to local conditions.

### Current Distribution of *P. notoginseng* Under Different Regionalization Models

**Figure 3:**
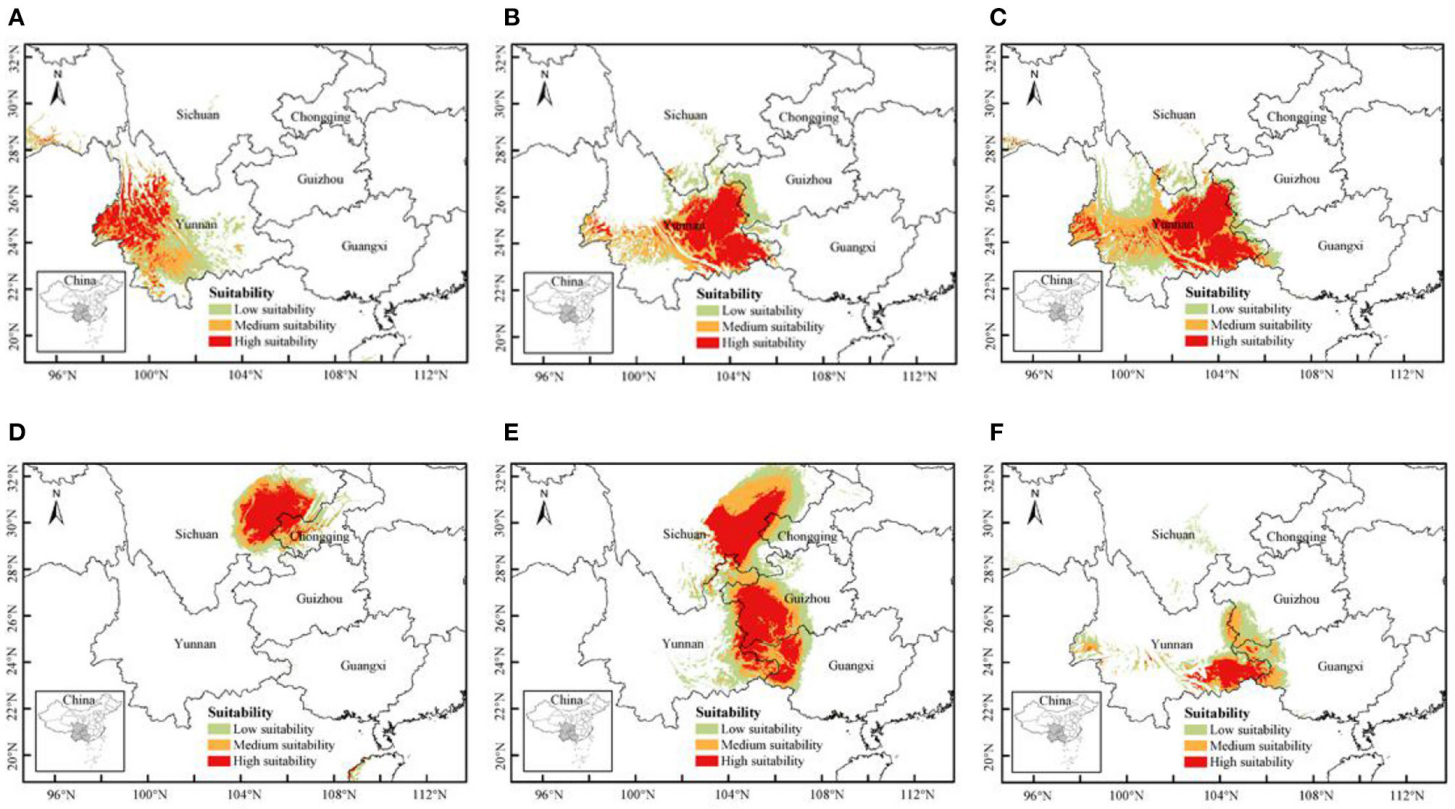
The potential distribution of *Panax notoginseng* under current climate based on different regionalization model. **(A)** Hengduan Mountain regionalization, **(B)** Yunnan-Guizhou Plateau regionalization, **(C)** Yunnan + Guangxi regionalization, **(D)** Sichuan Basin regionalization, **(E)** Sichuan + Chongqing + Guizhou regionalization, and **(F)** Sichuan + Chongqing + Guizhou + Guangxi + Wenshan regionalization.

MaxEnt model was used to predict the current potential distribution areas of *P. notoginseng* in different regionalization. The AUC values of the training set and test set data of the six models under different regionalization were all >0.920 (Supplementary Table S3), indicating that the model fitting effect was good and the prediction accuracy was high. The suitable habitat area data of the whole country and five provinces are shown in Supplementary Table S4, and the intuitive comparison chart is shown in Supplementary Figure S3. The distribution of suitable habitats for *P. notoginseng* in different regionalization models is shown in Figure 3.

Figure 3A shows the distribution of suitable habitat of *P. notoginseng* based on the regionalization of Hengduan Mountain. Compared to the overall regionalization model, the high suitable habitats in Hengduan Mountain region shifted and expanded to the northwest, and the area of low suitable habitats in Yunnan increased. Figure 3B shows the distribution of suitable habitat of *P. notoginseng* based on the regionalization model of Yunnan-Guizhou Plateau. Compared to the overall regionalization model, the regional change of high suitable habitat in Yunnan-Guizhou Plateau region is not significant, but the area of moderate and low suitable habitat is significantly reduced. Figure 3C shows the distribution of suitable habitat of *P. notoginseng* based on the regionalization of Yunnan + Guangxi. The regional distribution of high suitable habitat is roughly consistent with the overall regionalization model.

Figure 3D shows the suitable habitat distribution of *P. notoginseng* based on the regionalization of Sichuan Basin. Figure 3E shows the suitable habitat distribution of *P. notoginseng* based on Sichuan + Guizhou regionalization. The results of high, moderate, and low suitable habitat predicted by the two regionalization models were significantly different from those of the overall regionalization model. From the Figure 3 and Supplementary Table S4, we can see that the area of high suitable habitat increased significantly in Sichuan, Guizhou, and Guangxi. Based on the Sichuan Basin Regionalization model, the high suitable habitat area in Sichuan is 46 times more than that under the overall regionalization model. The area of high suitable habitat in Sichuan, Guangxi, and Guizhou based on the Sichuan + Guizhou regionalization model is 57 times, 91 times, and 104 times that of Sichuan, Guangxi, and Guizhou based on the overall regionalization model, respectively. Figure 3F shows the suitable habitat distribution of *P. notoginseng* based on the Sichuan + Guizhou + Guangxi + Wenshan regionalization model. This regionalization added Guangxi and Wenshan on the basis of Sichuan + Guizhou regionalization. The prediction results of the model are greatly different from the results of model in Figure 3E. The high suitable habitat area is mainly distributed in Wenshan, with an area of only 1.71 × 10^4^ km^2^.

In general, compared to the overall regionalization model, the distribution of high suitable habitat in the Yunnan-Guizhou Plateau regionalization model and Yunnan + Guangxi regionalization model was consistent, while the other four regionalization models (A, D, E, and F) had significant changes in both area and location. This was caused by the differences of topographic, geomorphic, and climatic factors in different regionalization, which indicated that selecting the suitable habitat in these four divisions should refer to the prediction results of each regionalization rather than the overall regionalization. From models D, E, and F, we can see that Sichuan, Guangxi, and Guizhou should theoretically have larger suitable habitat areas. Since the overall regionalization model is more focused on traditional production areas with more distribution points, it conceals important environmental factors of new production areas, and underestimates the potential and suitability of planting *P. notoginseng* in Sichuan, Guangxi, and Guizhou. It is worth noting that the reason why the suitable area of the F model is so different from that of the E model may be due to the difference in the altitude. Therefore, for areas with large differences in topography and geomorphology, modeling according to different regionalization may be more practical in guiding the selection of suitable habitats in different regions than the overall regionalization modeling. In this study, modeling based on different regionalization can predict a larger area of suitable habitat and provide more choices for the introduction of *P. notoginseng*. However, a more reasonable and practical regionalization study is needed to provide a solid foundation for the suitable habitat analysis of *P. notoginseng*.

### Future Distribution of *P. notoginseng* Under Overall Regionalization Model

**Figure 4:**
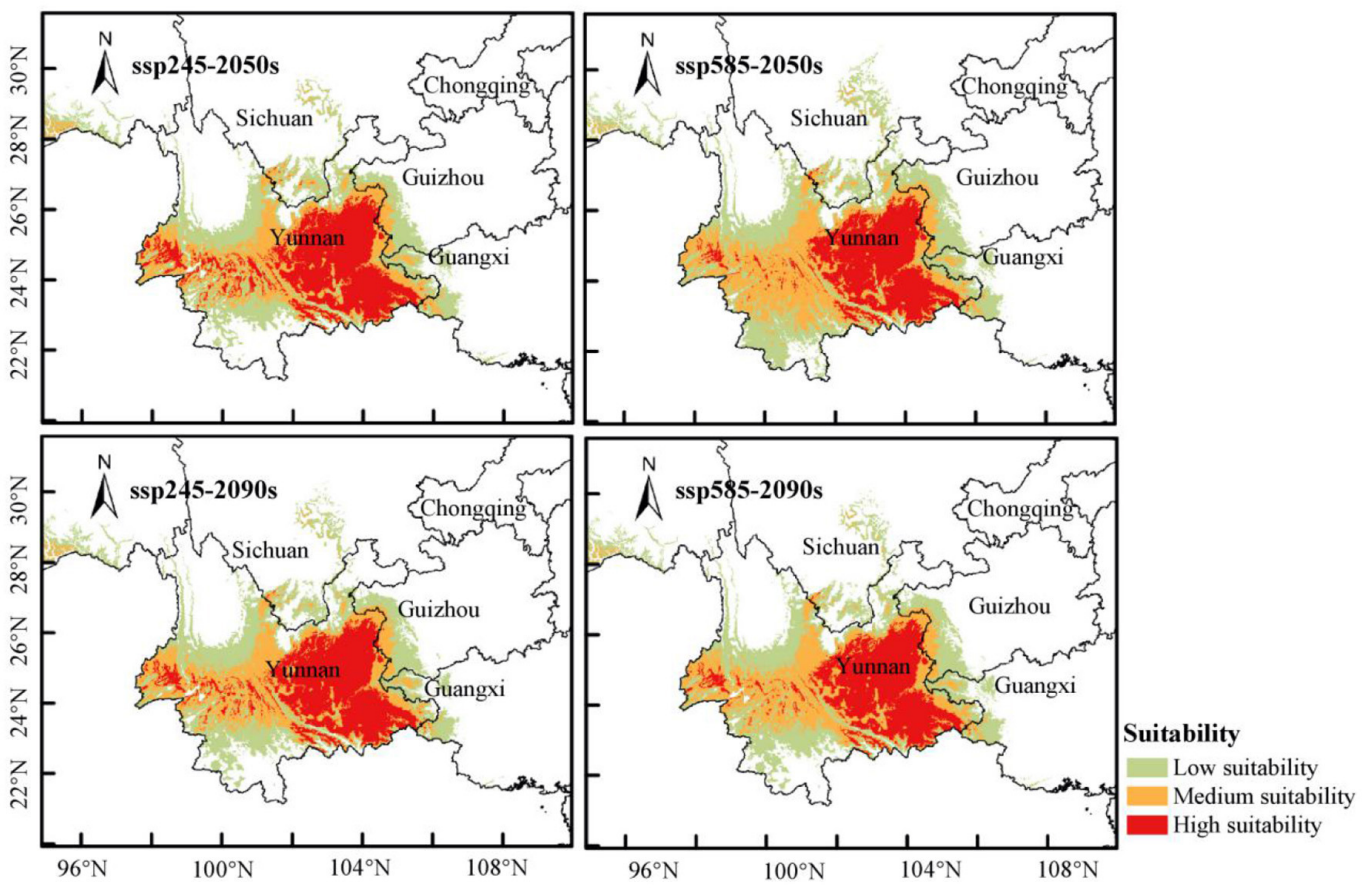
Future potential distribution of *Panax notoginseng* under climate change scenarios SSP245 and SSP585.

Under the background of climate change, two climate scenarios of 2050s and 2090s were selected respectively. The optimum MaxEnt model was used to simulate the geographical distribution of *P. notoginseng*, and the suitable habitat distribution (Figure 4) and suitable habitat area (Table 2) under different climate scenarios in different periods was obtained. In order to evaluate the distribution changes of suitable habitat of *P. notoginseng* under current and future climate conditions, we also calculated the SHCR (Table 2). The results show that the total suitable habitat areas for low, moderate, and total areas increased under the four scenarios in the two periods. Among them, the areas of low, moderate, and total suitable habitats under the SSP585-2050s climate background increased the most, and their areas were 14.70 × 10^4^ km^2^, 11.07 × 10^4^ km^2^, and 33.96 × 10^4^ km^2^. Compared to the current climate scenario, the area of high suitable habitats has increased by 56.38, 31.16, and 21.94%, respectively. It is worth noting that under different climate scenarios, the increase of low suitable habitat is the most significant, and the SHCR is higher than 40%. The area of high suitable habitat decreased, and the area of high suitable habitat was 8.95 × 10^4^ km^2^, 8.58 × 10^4^ km^2^, 8.19 × 10^4^ km^2^, and 8.02 × 10^4^ km^2^ under the four scenarios, respectively. Compared to the current climate, the area of high suitable habitat decreased by 10.59, 14.29, 18.18, and 19.88%, respectively. In the scenario of SSP585-2090s, *P. notoginseng* has the smallest area of high suitable area in the whole country.

**Table 2:** The distribution area and suitable habitat change rate of *Panax notoginseng* under future climate scenarios.

**Scenario**	**Area (×10^4^ km^2^)**				**SHCR (%)**			
	**L**	**M**	**H**	**T**	**L**	**M**	**H**	**T**
ssp245-2050s	12.40	8.90	8.95	30.25	31.91	5.45	−10.59	8.62
ssp245-2090s	13.59	9.34	8.58	31.51	44.57	10.66	−14.29	13.14
ssp585-2050s	14.70	11.07	8.19	33.96	56.38	31.16	−18.18	21.94
ssp585-2090s	13.65	9.90	8.02	31.57	45.21	17.30	−19.88	13.36

*L, Low suitable habitat; M, Moderate suitable habitat; H, High suitable habitat; T, Total suitable habitat; SHCR, Suitable habitat change rate*.

In this study, it was found that *P. notoginseng* has the widest distribution and the largest area of high suitable habitat under the current climate scenario. With the increase of time and emission intensity, the area of high suitable habitat decreased, and the emission intensity had a greater impact on the area of high suitable habitat than that of time. The higher the emission intensity in the same period, the more is the decrease in the area of high suitable habitat, indicating that the scenario of high concentration of CO_2_ emission had a greater impact on the potential distribution area of *P. notoginseng*. The area of high suitable habitat of *P. notoginseng* decreased in China and other provinces, indicating that the high suitable habitat of *P. notoginseng* will be degraded seriously under the background of future climate change. Yunnan was the province with the largest distribution of high suitable habitat area under different climate scenarios in different periods. The total suitable habitat area increased significantly, while the high suitable habitat area decreased, indicating that the high suitable habitat was transforming to the moderate and low suitable habitat, and the moderate and low suitable habitat was expanding continuously. The transformation and expansion of suitable habitat in Sichuan was consistent with that in Yunnan under different climate scenarios in different periods, while the total suitable habitat area in Guangxi and Guizhou decreased, and the moderate and low suitable habitat area showed slight expansion and contraction. The suitable habitat areas in Yunnan and Sichuan under different climate scenarios in various periods will continue to expand in the future, and the phenomenon with the period and emissions intensity had no obvious positive correlation, indicating that Yunnan is still the main area for the cultivation and expansion of *P. notoginseng* both at present and in the future, and Sichuan is the potential area of expansion in the future. But it should be noted that the introduction and cultivation should be adapted to local conditions. A small-scale introduction should be carried out first, and then a large-scale promotion can be carried out after successful trial planting. In addition, ecological planting modes based on regional distribution have gradually developed, such as intercropping of medicine and grain, cropping rotation and under-forest planting (Kang et al., 2020), which can effectively alleviate land shortage caused by continuous cropping obstacles under traditional planting mode, provide high-quality medicinal materials, and promote the development of multi-dimensional cultivation mode. These cropping patterns were coupled with species interaction and habitat demand for growing environment. However, climate change will affect species richness and community stability. Therefore, in addition to the prediction of a single species in the study of suitable habitats for *P. notoginseng* in the future climate, it is also necessary to coordinate the study of suitable habitats between regional communities.

### Quality Suitability Analysis

#### Quality Evaluation of Samples in High Suitable Habitat

The contents of notoginsenoside R_1_, ginsenoside Rg_1_, ginsenoside Re, ginsenoside Rb_1_, total flavonoids, and total polysaccharides of *P. notoginseng* samples from five high suitable habitats (Wenshan. Honghe, Yuxi, Kunming, and Qujing) were determined (Supplementary Table S5). Supplementary Figure S4 is the boxplot of content data. As can be seen from the Supplementary Figure S4 and Supplementary Table S5, the content of ginsenoside Rg_1_ is the highest, followed by ginsenoside Rb_1_, notoginsenoside R_1_ is the third, and the content of ginsenoside Re is the lowest among the four saponins. Among the five regions, notoginsenoside R_1_ and ginsenoside Rg_1_ in Honghe, ginsenoside Re, and total flavonoids in Wenshan, ginsenoside Rb_1_ in Yuxi, and total polysaccharides in Kunming were the highest. Taken together, the samples from Honghe had better quality than those from the other high suitable habitat according to the standard of “Pharmacopeia of People's Republic of China.” We speculated that the difference in content was caused by the elevation of the collection sites, changeable climatic conditions, or other natural factors, which led to the genetic variation of the samples or the change of their own metabolites.

In general, the contents of notoginseng R_1_, ginsenoside Rg_1_, and ginsenoside Rb_1_ in 50 samples of *P. notoginseng* were higher than the standard contents stipulated by Chinese Pharmacopeia of 2020 Edition (National Pharmacopoeia Committee, 2020), indicating that the *P. notoginseng* samples in the five high suitable habitats had high quality. It can be seen from the above results that the ecological suitability and quality suitability of *P. notoginseng* samples in the high suitable habitat are consistent. However, this study also has some problems, such as insufficient sample size and coverage of sample collection points in different regions, so further research is needed.

#### PCA Analysis Based on Content Data and Environmental Factors

Principal Component Analysis is a multivariate statistical technique, which simplifies data structure by simplifying multiple variables into a few comprehensive variables with the idea of dimensionality reduction. PCA analysis was carried out on the content data of *P. notoginseng* and corresponding environmental variables of different sampling sites in five regions. This study revealed the relationship between environmental factors and seven active ingredients using principal components. Supplementary Figure S5 is the scree plot of PCA, which is a graph of eigenvalues sorted from the largest to the smallest, used to determine the number of principal components. The first five principal components explained 92.3% of the variables cumulatively, of which 41.2% of the variables were explained by PC1. Supplementary Figure S6 is a loading plot for PCA, showing the contribution rate of each variable to the principal component. The red dashed line on the graph above indicates the expected average contribution. For a given component, a variable with a contribution larger than this cutoff could be considered as important in contributing to the component. Supplementary Figure S7 is a variable correlation plot colored by groups. It shows the relationships between all variables. It can be interpreted as follow: Positively correlated variables are grouped together. Negatively correlated variables are positioned on opposite sides of the plot origin (opposed quadrants). The distance between variables and the origin measures the quality of the variables on the factor map. Variables that are away from the origin are well-represented on the factor map.

Obviously, Bio12, Bio16, Bio18, and Bio13 have a positive effect on the accumulation of GB, GE, and NR, especially GB and GE. However, Bio07, Bio02, and Ele are not conducive to the accumulation of these three components, but they are conducive to the accumulation of TP. Bio14, Bio04, Bio19, and Bio17 are beneficial to the accumulation of TF, but not beneficial to the accumulation of NR and GR. There are many environmental variables that have a positive effect on GR accumulation, and all of them are distributed in the fourth quadrant. In particular, Bio05 has a significant effect on GR accumulation. The study results of Feng et al. (2006) on *P. notoginseng* showed that the precipitation in January and the annual temperature difference were the key factors affecting the total saponin of *P. notoginseng*. The precipitation was beneficial to the accumulation of flavonoid, but inhibited the accumulation of total saponin, polysaccharides, and dencichine in *P. notoginseng*. This is consistent with the correlation analysis results of this study. In addition, other related studies also showed that the region with small annual temperature difference was conducive to the accumulation of notoginseng saponin. Excessive precipitation would inhibit the accumulation of notoginseng saponin, while the overcast and rainy environment with more water was conducive to the accumulation of flavonoid, but not conducive to the accumulation of dencichine (Dong et al., 2003). Therefore, in the cultivation of *P. notoginseng*, appropriate artificial control of relevant environmental factors, such as light, humidity and so on, is conducive to the accumulation of important characteristic components of *P. notoginseng*.

### Rapid Identification of Samples From Different Suitable Habitat

In this study, ResNet models were established based on FT-MIR and NIR 2DCOS images to identify *P. notoginseng* samples from different suitable areas. The discriminative ability of all models was evaluated by accuracy and loss values. External validation was used to judge and evaluate the strengths and weaknesses of the models to ensure the stability of the established model.

**Figure 5:**
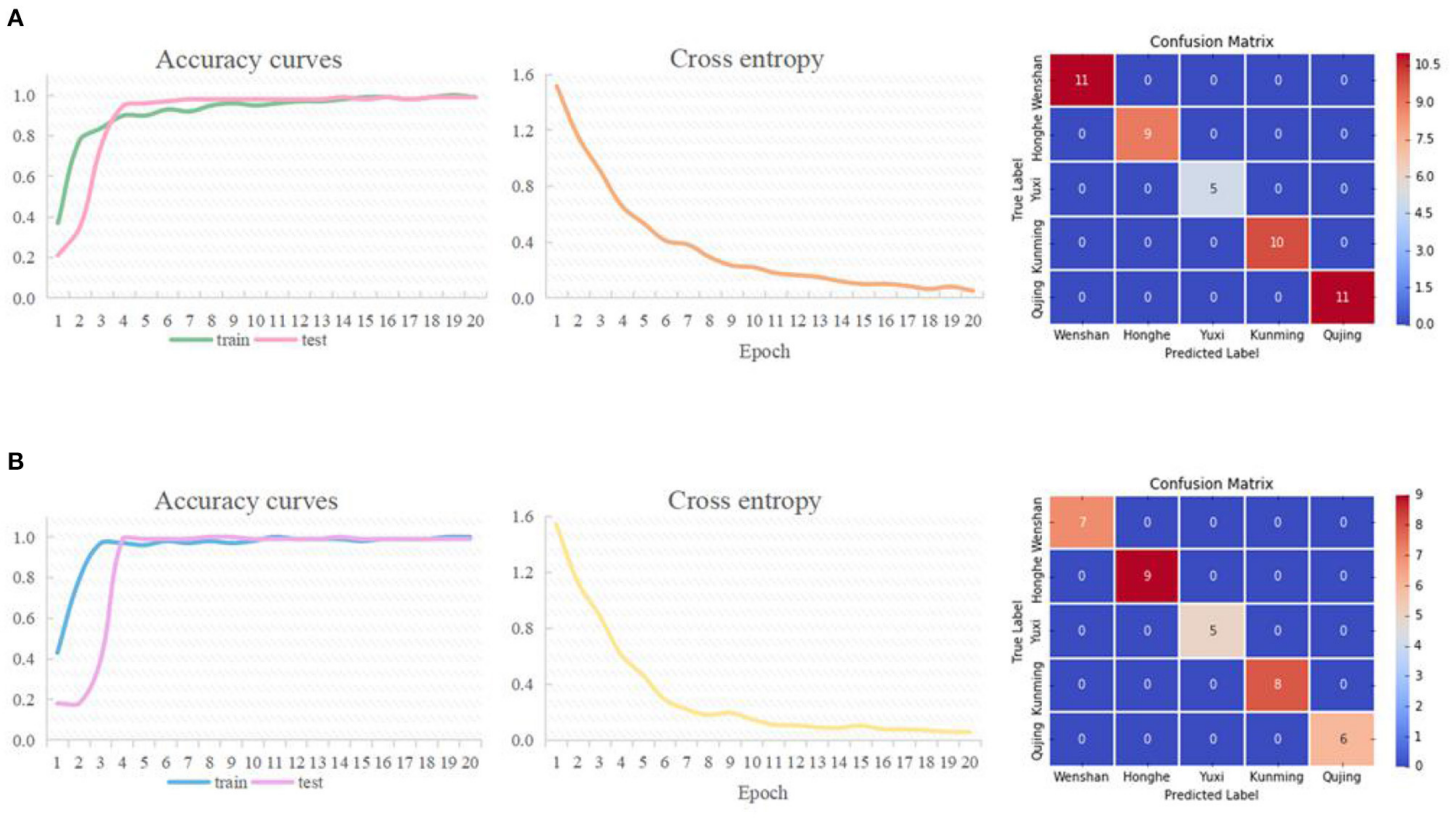
The accuracy curves, cross entropy, and confusion matrix of ResNet models based on different spectral data. **(A)** FT-MIR, **(B)** NIR.

Figure 5 shows the accuracy curve, cross entropy, and external verification results of the established models. Figure 5A is the identification result based on the FT-MIR 2DCOS images. The results show that the accuracy of the training set is 99%, the test set is 99%, the external validation set is 100%, the number of epochs is 20, and the loss value is 0.053. Figure 5B is the identification result based on the NIR 2DCOS images. The results show that the accuracy of the training set is 100%, test set is 99%, the external validation set is 100%, the number of epochs is 20, and the loss value is 0.056.

According to the modeling results, ResNet models based on FT-MIR and NIR data can achieve rapid identification of *P. notoginseng* samples from five suitable areas. From the perspective of accuracy rate, lost value, and external verification results, the two models have almost no difference and have excellent discrimination performance with almost 100% accuracy. This result may be related to the learning performance of the deep learning model itself, which can extract features of different images for learning and finally realize the identification of target objects (Bargshady et al., 2020). The accuracy curves based on the training set and test set are highly consistent with the increasing trend of training rounds, which indicates that the model has no over-fitting risk and good robustness. The loss values of these models are close to zero, which verifies that the established models have good convergence effect.

In conclusion, deep learning combined with 2DCOS is an efficient and simple recognition method, which eliminates the need to manually extract sample data features and reduces the interference of human factors. As a means of identification, it is applied after ecological suitability evaluation and quality suitability evaluation, so as to realize the rapid identification and evaluation of *P. notoginseng* samples with both ecological suitability and quality suitability.

## Conclusion

This study combined theory with practice to study the geographical distribution pattern of *P. notoginseng* under current and future climate scenarios and predicted the suitable habitat of *P. notoginseng* through overall regionalization model and different regionalization model. The ecological suitability and quality suitability of *P. notoginseng* samples were analyzed by integrating multi-source chemical information of samples from different suitable habitats with modern analytical methods, and the rapid identification method of high-quality samples from different suitable habitats was established. Under the overall regionalization model, *P. notoginseng* is mainly distributed in the southwest China, and its high suitable habitat areas are mainly concentrated in Yunnan, China. Under future climate scenarios, the high suitable habitat will be severely degraded, and greenhouse gas emissions will not be conducive to the distribution and diffusion of *P. notoginseng*. However, the suitable habitat areas in Yunnan and Sichuan are expanding continuously. Whether at present or in the future, Yunnan is still the main area for the cultivation and expansion of *P. notoginseng*, and Sichuan is a region with a great potential for the expansion of *P. notoginseng* in the future. Modeling based on different regionalization can more accurately predict suitable habitats in a smaller area. Especially in Sichuan, Guizhou, Guangxi, and other places, the suitable habitat under different regionalization model is much higher than that of the overall regionalization model, which can provide more choices for the introduction and cultivation of *P. notoginseng*. The analysis of multi-source chemical information shows that the samples of *P. notoginseng* in the high suitable habitat have higher quality, and the ecological suitability and quality suitability are consistent. In addition, changes in habitat will also affect the quality of medicinal materials. The accumulation of saponin, polysaccharide, and flavonoid in the samples is affected by various environmental factors. Proper regulation of relevant environmental factors during the planting process is conducive to the improvement of the important characteristic components of *P. notoginseng*. Deep learning combined with 2DCOS can realize the identification of samples in different suitable areas, providing an efficient and simple method for the evaluation of Traditional Chinese medicine. This study can guide the reasonable layout of production base, and the introduction and breeding, and promote the sustainable development of high-quality *P. notoginseng* industry.

## Data Availability Statement

The original contributions presented in the study are included in the article/Supplementary Materials, further inquiries can be directed to the corresponding authors.

## Author Contributions

JY: conceptualization, methodology, software, validation, formal analysis, and writing-original draft preparation. ZL: resources, validation, and writing-review and editing. ZZ: supervision, project administration, and funding acquisition. YW: resources, supervision, project administration, and funding acquisition. All authors have read and agreed to the published version of the manuscript.

## Funding

This work was supposed by National Natural Science Foundation of China (Grant Number: 31760360), Natural Science Foundation of Yunnan Province of China (Grant Number: 202101AT070260), and Special Program for the Major Science and Technology Projects of Yunnan Province (Grant Number: 202002AA100007).

## Conflict of Interest

The authors declare that the research was conducted in the absence of any commercial or financial relationships that could be construed as a potential conflict of interest.

## Publisher's Note

All claims expressed in this article are solely those of the authors and do not necessarily represent those of their affiliated organizations, or those of the publisher, the editors and the reviewers. Any product that may be evaluated in this article, or claim that may be made by its manufacturer, is not guaranteed or endorsed by the publisher.
