# Supplementary material for: Evaluation of Ecological Suitability and Quality Suitability of Panax notoginseng Under Multi-Regionalization Modeling Theory
Source: Front Plant Sci. 2022 Apr 28;13:818376. doi: 10.3389/fpls.2022.818376 (PMC9096839; doi:10.3389/fpls.2022.818376)
Supplement: Supplementary file 1 [file Data_Sheet_1.docx]

**Supplementary materials**

**Figures**

Figure S1 The structure of ResNet model used in this research.

Figure S2 The identification flow chart of ResNet model.

Figure S3 The suitable habitat data *Panax notoginseng* based on seven regionalization models.

Figure S4 The box-plot of content data.

Figure S5 The scree plot of principal component analysis.

Figure S6 The loading plot of principal component analysis result.

**Tables**

Table S1 The detailed distribution data of *Panax notoginseng* and number of samples used for content analysis and spectral analysis.

Table S2 ResNet network parameter configuration.

Table S3 The AUC values and important environmental variables of seven models based on different regionalization under current climate conditions.

Table S4 The suitable habitat area of *Panax notoginseng* based on seven regionalization models.

Table S5 The content information of *Panax notoginseng* in five suitable habitat.


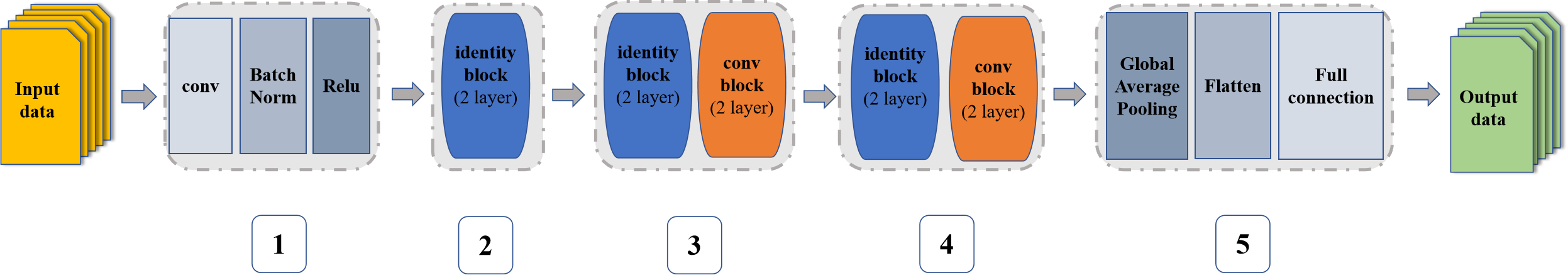


Figure S1 The structure of ResNet model used in this research.


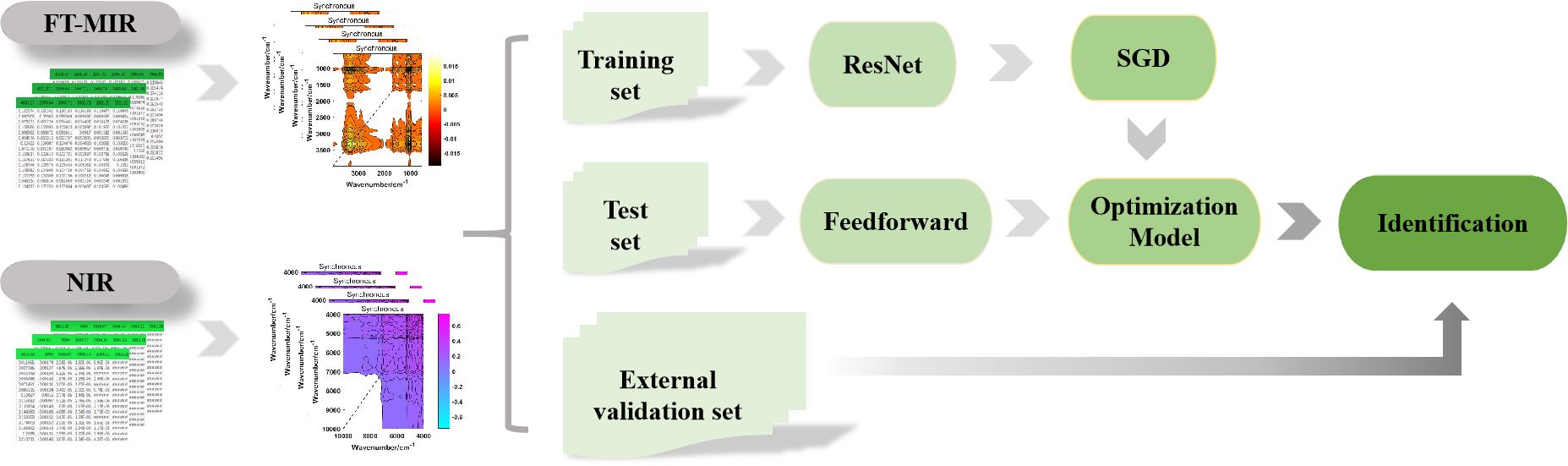


Figure S2 The identification flow chart of ResNet model.


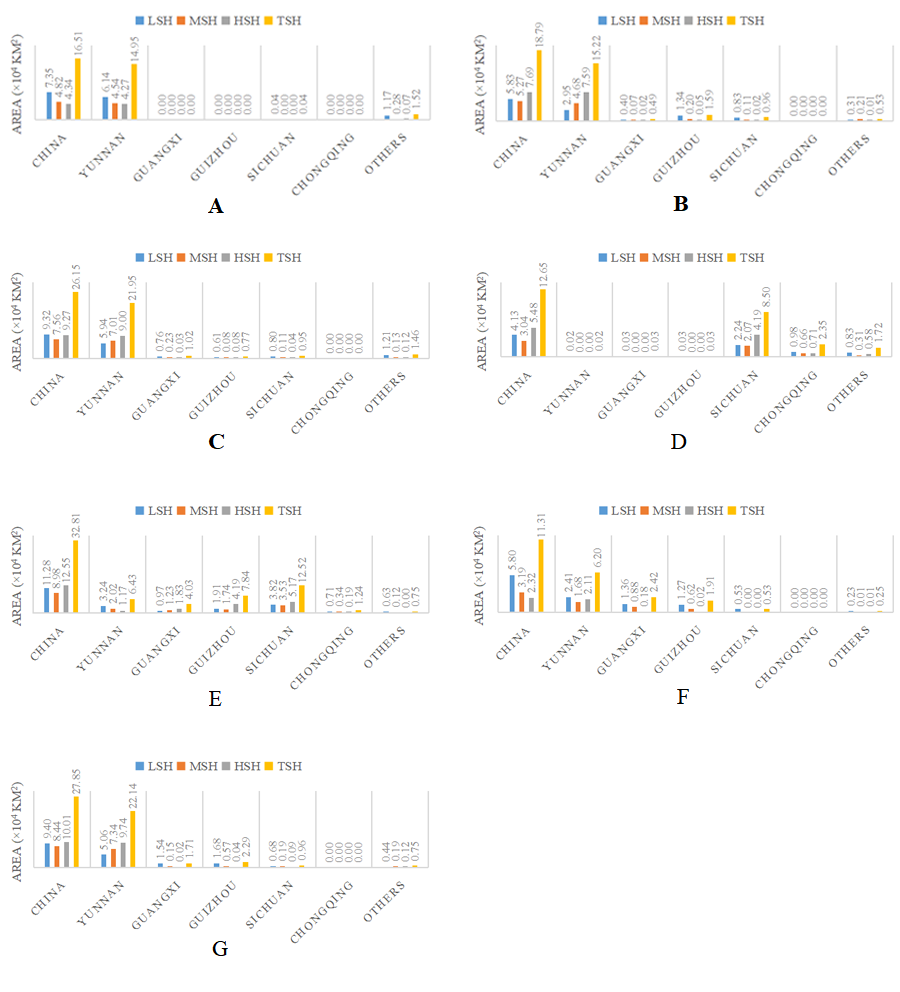


Figure S3 The suitable habitat data *Panax notoginseng* based on seven regionalization models.

(A) Hengduan Mountain regionalization, (B) Yunnan-Guizhou Plateau regionalization, (C) Yunnan + Guangxi regionalization, (D) Sichuan Basin regionalization, (E) Sichuan + Chongqing + Guizhou regionalization, (F) Sichuan + Chongqing + Guizhou + Guangxi + Wenshan regionalization and (G) overall regionalization.


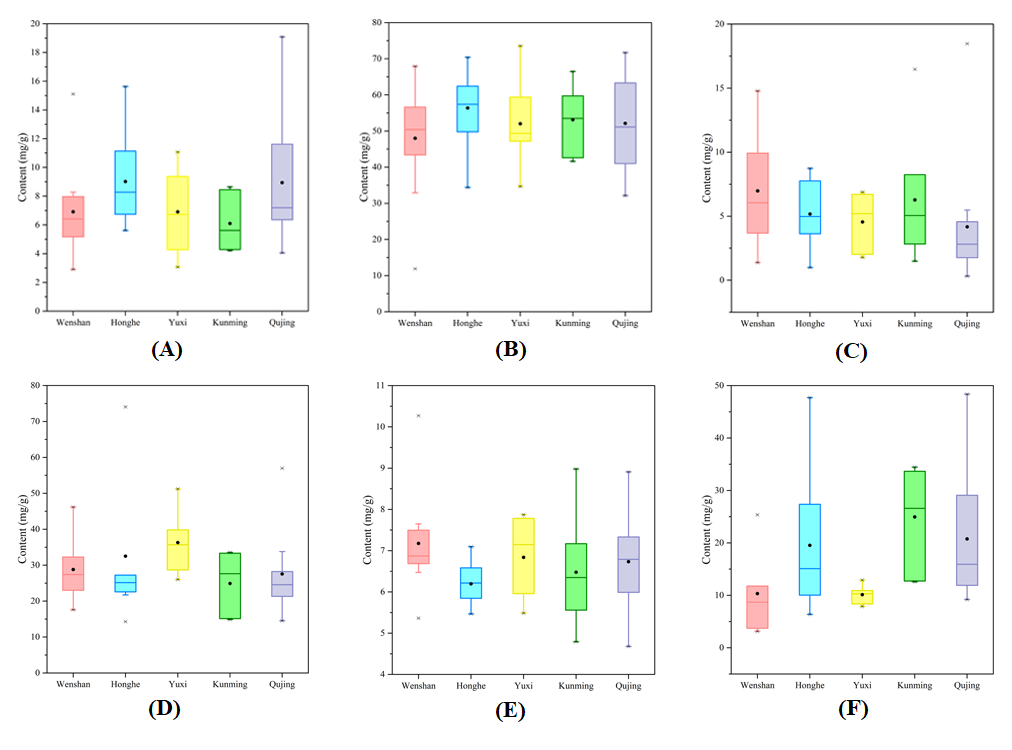


Figure S4 The box-plot of content data.

(A) notoginsenoside R_1_, (B) ginsenoside Rg_1_, (C) ginsenoside Re, (D) ginsenoside Rb_1_, (E) total flavonids and (F) total polysaccharides.


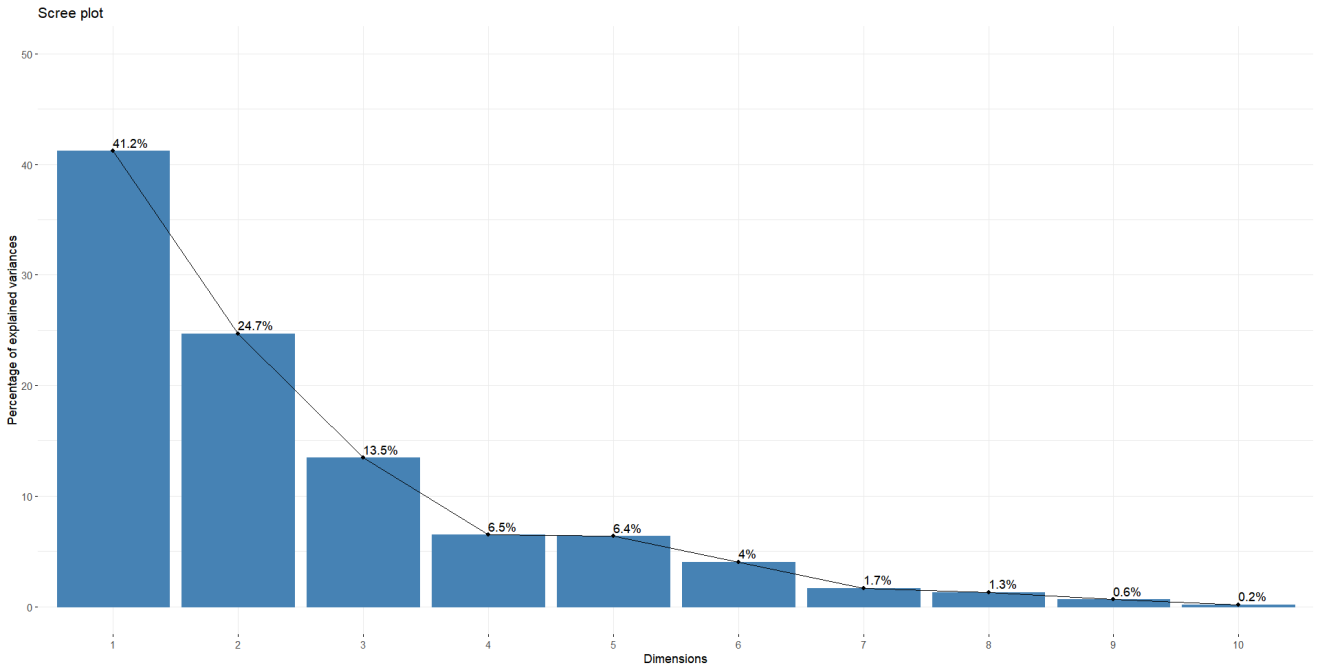


Figure S5 The scree plot of principal component analysis.


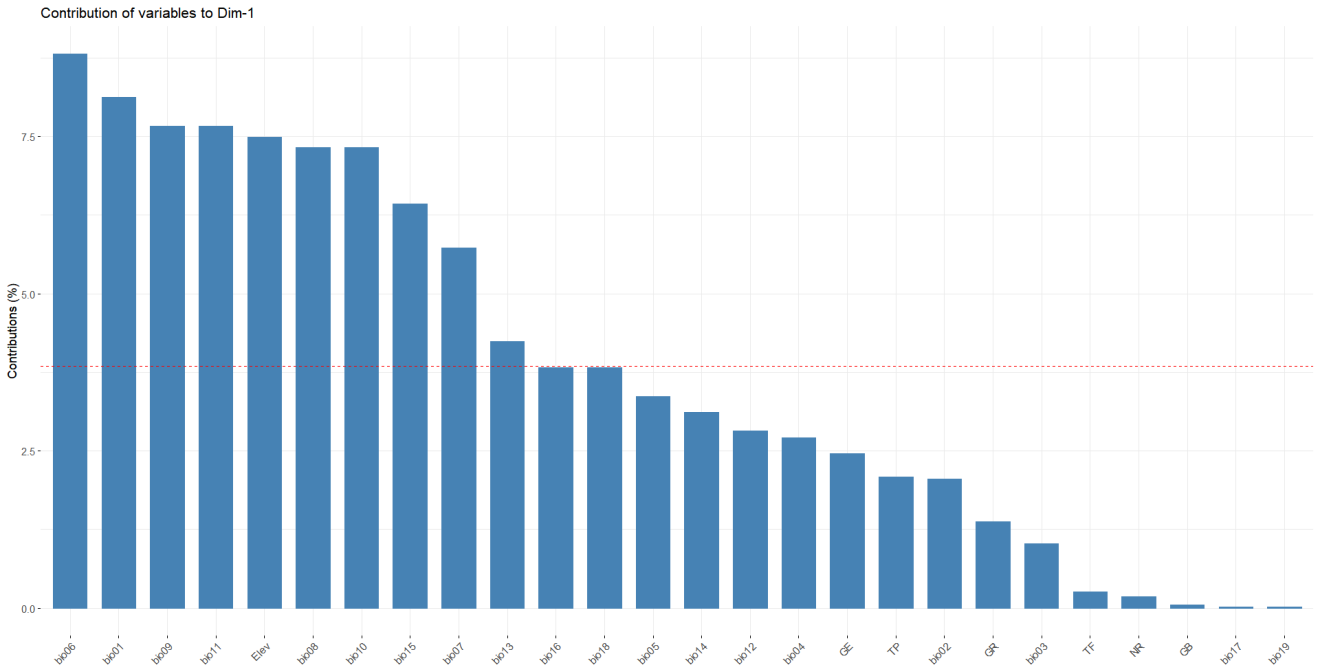


Figure S6 The loading plot of principal component analysis result.


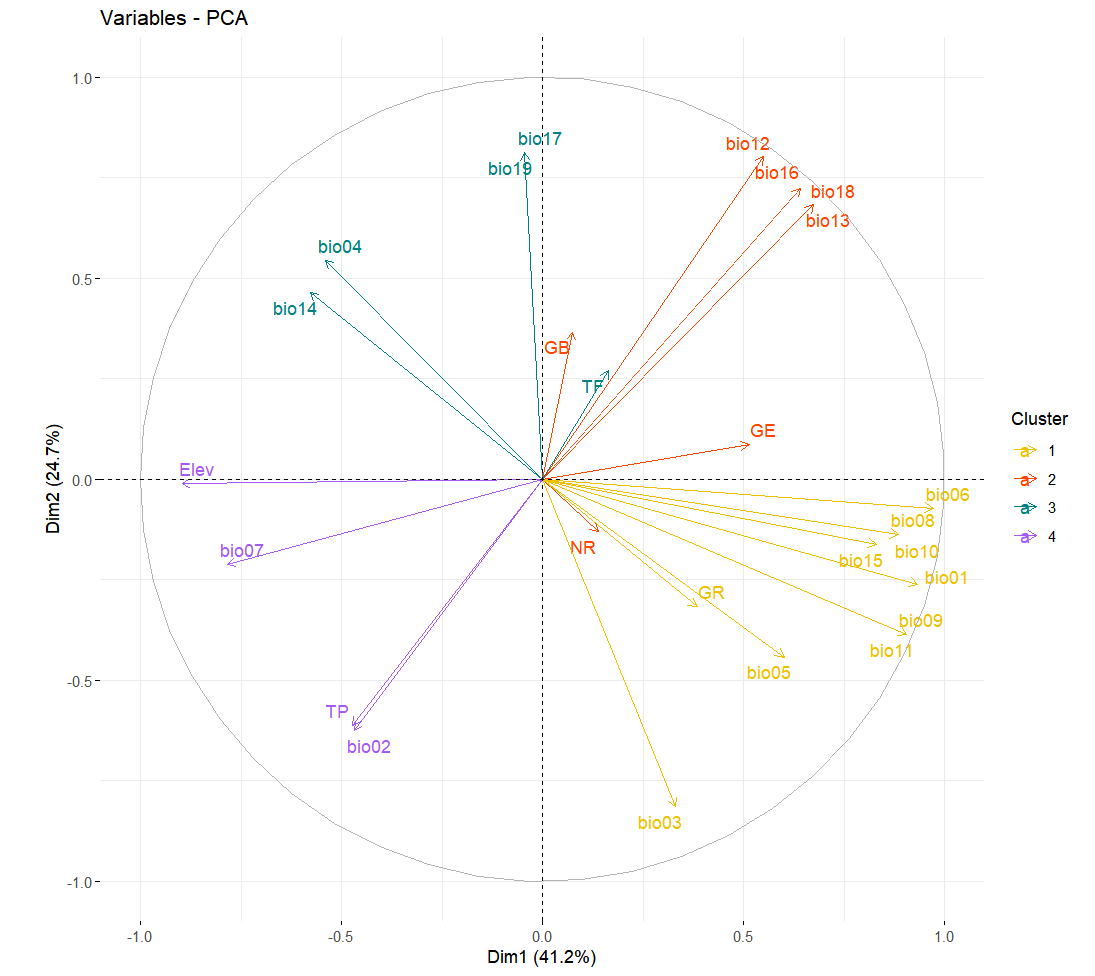


Figure S7 Variable correlation plot of principal component analysis analysis result.

Table S1 The detailed distribution data of *Panax notoginseng* and number of samples used for content analysis and spectral analysis.

| Code | Province | Region | NL | sample size | | |
| --- | --- | --- | --- | --- | --- | --- |
|  |  |  |  | Content | FT-MIR | NIR |
| 1 | Guangxi | Baise | 22 | / | / | / |
| 2 | Guizhou | Anshun | 2 | / | / | / |
|  |  | Liupanshui | 4 | / | / | / |
|  |  | Qiannan | 1 | / | / | / |
|  |  | Qianxinan | 14 | / | / | / |
| 3 | Sichuan | Nanchong | 2 | / | / | / |
|  |  | Suining | 7 | / | / | / |
| 4 | Chongqing | Rongchang | 1 | / | / | / |
|  |  | Tongnan | 1 | / | / | / |
| 5 | Yunnan | Baoshan | 39 | / | / | / |
| 6 |  | Chuxiong | 33 | / | / | / |
| 7 |  | Dali | 7 | / | / | / |
| 8 |  | Dehong | 10 | / | / | / |
| 9 |  | **Honghe** | **673** | **10** | **90** | **89** |
| 10 |  | **Kunming** | **282** | **7** | **97** | **80** |
| 11 |  | Lijiang | 9 | / | / | / |
| 12 |  | Puer | 12 | / | / | / |
| 13 |  | **Qujing** | **351** | **15** | **109** | **63** |
| 14 |  | **Wenshan** | **524** | **11** | **110** | **73** |
| 15 |  | Xishuangbanna | 1 | / | / | / |
| 16 |  | **Yuxi** | **123** | **7** | **49** | **49** |
| 17 |  | Lincang | 4 | / | / | / |
| Total | / | / | 2122 | 50 | 455 | 354 |

Note: Number of locations, NL; Bold indicates the area with more distribution points, where samples are used for subsequent chemical analysis.

Table S2 ResNet network parameter configuration.

| Layer name | Kernel size | Channels | Strides | Block numbers | Block name |
| --- | --- | --- | --- | --- | --- |
| Conv1 | 3×3 | 32 | 1 | ×1 | Identity Block |
|  |  |  |  |  |  |
| Conv2_x | 3×3 | 32 | 1 | ×1 | Identity Block |
|  | 3×3 | 32 | 1 |  |  |
|  |  |  |  |  |  |
| Conv3_x | 3×3 | 32 | 1 | ×1 | Identity Block |
|  | 3×3 | 32 | 1 |  |  |
|  |  |  |  |  |  |
|  | 3×3 | 64 | 2 | ×1 | Conv Block |
|  | 3×3 | 64 | 1 |  |  |
|  | 1×1 | 64 | 2 |  |  |
|  |  |  |  |  |  |
| Conv4_x | 3×3 | 64 | 1 | ×1 | Identity Block |
|  | 3×3 | 64 | 1 |  |  |
|  |  |  |  |  |  |
|  | 3×3 | 128 | 2 | ×1 | Conv Block |
|  | 3×3 | 128 | 1 |  |  |
|  | 1×1 | 128 | 2 |  |  |
|  |  |  |  |  |  |
| Output | Global average pooling, Flatten, Full Connection, Softmax | | | | |

Table S3 The AUC values and important environmental variables of seven models based on different regionalization under current climate conditions.

| Model | Training | Test | Bio 02 | Bio 03 | Bio 04 | Bio 07 | Bio 13 | Bio 14 | Bio 15 | Bio17 | Bio 18 | Bio 19 | Elev |
| --- | --- | --- | --- | --- | --- | --- | --- | --- | --- | --- | --- | --- | --- |
| 1 | 0.993 | 0.992 |  | √√√√ | √√√√√ |  |  | √√√ |  | √√ |  |  | √ |
| 2 | 0.932 | 0.932 | √√√ |  | √√√√√ |  |  | √ |  | √√ |  |  | √√√√ |
| 3 | 0.929 | 0.928 |  |  | √√√ | √√ |  |  |  | √ |  |  |  |
| 4 | 0.997 | 0.997 |  | √√ | √√√√ |  |  |  |  |  |  | √ | √√√ |
| 5 | 0.994 | 0.991 | √√√ |  |  |  |  | √√√√√ | √√√√ |  | √√√√√√ | √ | √√ |
| 6 | 0.978 | 0.978 |  |  | √√√√√ | √ | √√√ |  | √√ | √√√√√√ |  |  | √√√√ |
| 7 | 0.927 | 0.927 |  |  | √√√√ | √√√ |  |  |  | √ |  |  | √√ |

Note: the number of “√” indicates the importance of different important environmental variables under the same model. The more the number, the higher the importance.

Table S4 The suitable habitat area of *Panax notoginseng* based on seven regionalization models.

| Model | Suitable habitat | China | Yunnan | Guangxi | Guizhou | Sichuan | Chongqing | Others |
| --- | --- | --- | --- | --- | --- | --- | --- | --- |
| 1 | L | 7.35 | 6.14 | 0.00 | 0.00 | 0.04 | 0.00 | 1.17 |
|  | M | 4.82 | 4.54 | 0.00 | 0.00 | 0.00 | 0.00 | 0.28 |
|  | H | 4.34 | 4.27 | 0.00 | 0.00 | 0.00 | 0.00 | 0.07 |
|  | T | 16.51 | 14.95 | 0.00 | 0.00 | 0.04 | 0.00 | 1.52 |
| 2 | L | 5.83 | 2.95 | 0.40 | 1.34 | 0.83 | 0.00 | 0.31 |
|  | M | 5.27 | 4.68 | 0.07 | 0.20 | 0.11 | 0.00 | 0.21 |
|  | H | 7.69 | 7.59 | 0.02 | 0.05 | 0.02 | 0.00 | 0.01 |
|  | T | 18.79 | 15.22 | 0.49 | 1.59 | 0.96 | 0.00 | 0.53 |
| 3 | L | 9.32 | 5.94 | 0.76 | 0.61 | 0.80 | 0.00 | 1.21 |
|  | M | 7.56 | 7.01 | 0.23 | 0.08 | 0.11 | 0.00 | 0.13 |
|  | H | 9.27 | 9.00 | 0.03 | 0.08 | 0.04 | 0.00 | 0.12 |
|  | T | 26.15 | 21.95 | 1.02 | 0.77 | 0.95 | 0.00 | 1.46 |
| 4 | L | 4.13 | 0.02 | 0.03 | 0.03 | 2.24 | 0.98 | 0.83 |
|  | M | 3.04 | 0.00 | 0.00 | 0.00 | 2.07 | 0.66 | 0.31 |
|  | H | 5.48 | 0.00 | 0.00 | 0.00 | 4.19 | 0.71 | 0.58 |
|  | T | 12.65 | 0.02 | 0.03 | 0.03 | 8.50 | 2.35 | 1.72 |
| 5 | L | 11.28 | 3.24 | 0.97 | 1.91 | 3.82 | 0.71 | 0.63 |
|  | M | 8.98 | 2.02 | 1.23 | 1.74 | 3.53 | 0.34 | 0.12 |
|  | H | 12.55 | 1.17 | 1.83 | 4.19 | 5.17 | 0.19 | 0.00 |
|  | T | 32.81 | 6.43 | 4.03 | 7.84 | 12.52 | 1.24 | 0.75 |
| 6 | L | 5.80 | 2.41 | 1.36 | 1.27 | 0.53 | 0.00 | 0.23 |
|  | M | 3.19 | 1.68 | 0.88 | 0.62 | 0.00 | 0.00 | 0.01 |
|  | H | 2.32 | 2.11 | 0.18 | 0.02 | 0.00 | 0.00 | 0.01 |
|  | T | 11.31 | 6.20 | 2.42 | 1.9 | 0.53 | 0.00 | 0.25 |
| **7** | L | 9.40 | 5.06 | 1.54 | 1.68 | 0.68 | 0.00 | 0.44 |
|  | M | 8.44 | 7.34 | 0.15 | 0.57 | 0.19 | 0.00 | 0.19 |
|  | H | 10.01 | 9.74 | 0.02 | 0.04 | 0.09 | 0.00 | 0.12 |
|  | T | 27.85 | 22.14 | 1.71 | 2.29 | 0.96 | 0.00 | 0.75 |

Note: 1, Hengduan Mountain regionalization model; 2, Yunnan-Guizhou Plateau regionalization model; 3, Yunnan+Guangxi regionalization model; 4, Sichuan Basin regionalization model; 5. Sichuan+Chongqing+Guizhou regionalization model; 6, Sichuan+Chongqing+Guizhou+Guangxi+Wenshan regionalization model; 7, overall regionalization model; L, low suitable habitat; M, moderate suitable habitat; H, high suitable habitat.

Table S5 The content information of *Panax notoginseng* in five suitable habitat.

| Code | Origin | notoginsenoside R_1_（mg/g） | ginsenoside Rg_1_（mg/g） | ginsenoside Re（mg/g） | ginsenoside Rb_1_（mg/g） | total flavonids（mg/g） | total polysaccharides（mg/g） |
| --- | --- | --- | --- | --- | --- | --- | --- |
| 1 | Wenshan | 6.92±3.17 | 47.98±15.17 | 6.97±3.98 | 28.80±8.63 | 7.17±1.20 | 10.34±7.86 |
| 2 | Honghe | 9.02±3.36 | 56.38±10.25 | 5.17±2.65 | 32.52±19.92 | 6.19±0.51 | 19.53±12.91 |
| 3 | Yuxi | 6.91±2.96 | 52.02±11.95 | 4.55±2.09 | 36.28±8.20 | 6.83±0.91 | 10.13±1.68 |
| 4 | Kunming | 6.09±1.80 | 53.12±9.00 | 6.27±5.03 | 24.90±7.88 | 6.47±1.33 | 24.95±9.51 |
| 5 | Qujing | 8.93±4.31 | 52.11±12.04 | 4.16±4.52 | 27.55±11.27 | 6.73±1.06 | 20.75±12.15 |
